# Safety and Pharmacokinetics of a Four Monoclonal Antibody Combination against Botulinum C and D Neurotoxins

**DOI:** 10.1128/AAC.01270-19

**Published:** 2019-11-21

**Authors:** Doris M. Snow, Kathryn Riling, Angie Kimbler, Yero Espinoza, David Wong, Khanh Pham, Zachary Martinez, Carl N. Kraus, Fraser Conrad, Consuelo Garcia-Rodriguez, Ronald R. Cobb, James D. Marks, Milan T. Tomic

**Affiliations:** a Ology Bioservices, Inc., Frederick, Maryland, USA; b Ology Bioservices, Inc., Alachua, Florida, USA; c Ology Bioservices, Inc., Berkeley, California, USA; d Arrevus, Raleigh, North Carolina, USA; e Department of Anesthesia and Perioperative Care, University of California, San Francisco, San Francisco, California, USA

**Keywords:** botulinum neurotoxin, safety, pharmacokinetics, monoclonal antibody, antibody combinations, phase 1 clinical trial, antitoxin, oligoclonal antibodies, recombinant antibodies

## Abstract

Botulism is caused by botulinum neurotoxin (BoNT), the most poisonous substance known. BoNTs are also classified as tier 1 biothreat agents due to their high potency and lethality. The existence of seven BoNT serotypes (A to G), which differ by 35% to 68% in amino acid sequences, necessitates the development of serotype-specific countermeasures. We present results of a phase 1 clinical study of an anti-toxin to BoNT serotypes C and D, NTM-1634, which consists of an equimolar mixture of four fully human IgG1 monoclonal antibodies (MAbs), each binding to nonoverlapping epitopes on BoNT serotypes C and D, resulting in potent toxin neutralization in rodents. This first in-human study evaluated the safety and pharmacokinetics of escalating doses of NTM-1634 administered intravenously to healthy adults. Three cohorts of eight healthy subjects received single intravenous doses of NTM-1634 at 0.33 mg/kg, 0.66 mg/kg, or 1 mg/kg or placebo. Follow-up examinations and pharmacokinetics evaluations were continued up to 121 days postinfusion. Subjects were monitored by using physical examinations, hematology and chemistry blood tests, and electrocardiograms. Pharmacokinetics parameters were estimated using noncompartmental methods. The results demonstrated that the materials were safe and well tolerated with the expected half-lives for human MAbs and with minimal antidrug antibodies detected over the dose ranges and duration of the study. (This study has been registered at ClinicalTrials.gov under identifier NCT03046550.)

## Abstract (precis)

Botulism is caused by botulinum neurotoxin (BoNT), the most poisonous substance known. BoNTs are also classified as tier 1 biothreat agents due to their high potency and lethality. The existence of seven BoNT serotypes (A to G), which differ by 35% to 68% in amino acid sequences, necessitates the development of serotype-specific countermeasures.

## INTRODUCTION

Botulism, an acute life-threatening flaccid paralysis affecting both humans and animals, is caused by botulinum neurotoxins (BoNTs) produced by the bacterium Clostridium botulinum and additional *Clostridia* species (1, 2). Naturally occurring botulism is an orphan disease, with approximately 120 cases/year in the United States. BoNTs are also classified as tier 1 biothreat agents, the highest level of classification, due to their high potency and lethality (3). As such, the U.S. government has funded the development of botulinum antitoxins, including those reported here.

Of the seven immunologically distinct types of BoNTs (A to G) (4–6), serotypes A, B, E, and F cause most of the naturally occurring human disease, including foodborne, wound, and intestinal botulism. Sequence analysis of BoNT/C and BoNT/D strains reveals the existence of mosaic toxins that contain portions of both BoNT/C and BoNT/D as well as sequences unique to the mosaics (7, 8). BoNT C/D has the sequence of BoNT/C for the amino-terminal two-thirds of the toxin but is 95% identical to the sequence of BoNT/D for the carboxy-terminal one-third. BoNT D/C has high identity with the BoNT/D amino terminus but shares a lower identity with the BoNT/C and BoNT/D carboxy termini (8). BoNT/C and BoNT/D most frequently intoxicate nonhumans, with BoNT/C and C/D causing botulism in avian species (9, 10) as well as feline and canine species; BoNT/D and D/C most frequently cause botulism in cattle (11, 12). BoNT/C and BoNT/D, however, can also cause botulism in humans (13). Two cases of foodborne botulism and one case of infant botulism have been attributed to BoNT/C (14). BoNT/D organisms have also been found in tainted ham that caused botulism in several individuals (15). BoNT/C blocks neuromuscular transmission in human neuromuscular junction preparations and causes prolonged inhibition of exocytosis in cerebellar granular neurons. Both BoNT/C and BoNT D/C cause lethal botulism in nonhuman primates exposed via the aerosol route. Finally, BoNT C/D is therapeutically active in treating dystonia in humans (16). These studies indicate that BoNT/C and BoNT/D and their mosaic toxins pose a similar biothreat as other BoNT serotypes. Thus, the development of countermeasures for all seven serotypes is a high priority of the National Institute for Allergy and Infectious Diseases (NIAID) and the Department of Health and Human Services (17).

The only treatment for botulism is antitoxin. As a result, the Public Health Emergency Medical Countermeasure Enterprise (PHEMCE) has a requirement for polyclonal BoNT antitoxin for the national stockpile for intentional botulism (17). The current treatment for adult botulism is heptavalent (serotypes A to G) equine botulism antitoxin (BAT) (18). BAT is immunogenic, and hypersensitivity reactions have been reported, including serum sickness and asystole (18). BAT is an F(ab′)_2_ product with short serum half-lives (7.5 to 34.2 h), which eliminates its use for prevention of botulism and limits its effectiveness as a treatment. Relapses of human botulism after treatment have been noted presumably due to the short half-life of BAT and poorer potency against some BoNT subtypes (19). BAT requires slow intravenous (i.v.) infusion after dilution into a total volume of 200 ml. This combined with hypersensitivity reactions makes it a challenge to administer in mass casualty scenarios. As an alternative, we have been developing serotype-specific monoclonal antibody combinations (three monoclonal antibodies [MAbs]/serotype) that potently and neutralize BoNT by eliciting first-pass clearance through the liver. Combining the three MAbs increases the potency of BoNT/C neutralization by at least 3 orders of magnitude over that of individual antibodies (C. Garcia-Rodriguez, unpublished data). In addition, we have reported the generation of three-MAb combinations to BoNT/A (20–22), BoNT/B (23), BoNT/E (24), BoNT/F (25), and BoNT/H (26) that are effective in mouse models of botulism and where the three-MAb combinations are more potent than single MAbs by 2 to 3 orders of magnitude. The products for botulism due to serotype A (NTM-1631, formerly XOMA 3AB) have completed a phase 1 clinical trial with no serious adverse effects (27). Phase 1 clinical trials for three-antibody combinations to treat botulism due to BoNT/B (ClinicalTrials registration number NCT02779140) and BoNT/E (ClinicalTrials registration number NCT03603665) are ongoing.

For the treatment and prevention of BoNT/C and BoNT/D botulism, we have developed a four-MAb combination, NTM-1634, that consists of an equimolar coformulated mixture of four fully human IgG1 monoclonal antibodies (MAbs), all with the same constant regions, referred to as XCD-a, XCD-b, XCD-c, and XCD-d. These MAbs bind nonoverlapping epitopes on BoNT/C, BoNT/D, and their mosaic toxins with high affinity and neutralize each of these BoNTs in the mouse neutralization assay. Here, we report the results of a phase 1, single-center, placebo-controlled, double-blind, randomized dose escalation study whose purpose was to evaluate the safety and pharmacokinetics properties of NTM-1634.

## RESULTS

### Antibody characteristics.

NTM-1634 consists of four human MAbs isolated from yeast display libraries constructed from the antibody variable region genes of humans immunized with pentavalent botulinum toxoid (C. Garcia-Rodriquez et al, unpublished data). Each MAb binds a nonoverlapping BoNT epitope with high affinity (Table 1). Three of the MAbs bind all four BoNTs with an equilibrium dissociation constant (*K_D_*) of <10^−9^ M, while MAb XCD-b only binds BoNT/C and BoNT D/C. A combination of the four parental MAbs from which MAbs XCD-a, XCD-b, XCD-c, and XCD-d were derived had effective doses that protected 50% of mice (ED_50_s) challenged with 40,000 50% lethal doses (LD_50_s) of BoNT/C of 5.0 μg/mouse, of BoNT C/D of 7.5 μg/mouse, and of BoNT D/C of 7.5 μg/mouse (C. Garcia-Rodriquez et al, unpublished data).

**TABLE 1:** Characteristics of the human monoclonal antibodies that comprise NTM-1634

Antibody name	No. of amino acids	Mol wt (kDa)	*K_D_* (10^−12^ M)			
			BoNT/C	BoNT C/D	BoNT D/C	BoNT/D
XCD-a	1,332	145,899	1.10	2.43	15.34	16.93
XCD-b	1,338	146,476	35.79	NB^*a*^	0.80	NB
XCD-c	1,338	146,463	11.79	37.81	83.95	96.20
XCD-d	1,334	145,788	130.37	37.90	22.44	11.63

a NB, no detectable binding at a BoNT concentration of 1 μM.

### Subject demographic characteristics.

**FIG 1:**
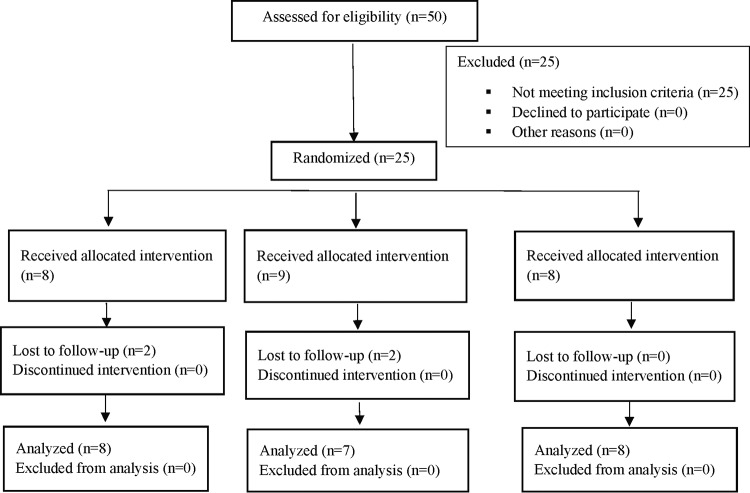
Consolidated Standards of Reporting Trials (CONSORT) diagram of clinical study design.

The use of 24 research participants was planned for these studies (Fig. 1). Twenty-five were enrolled, randomized, and included in the study. Randomized participants were administered either the NTM-1634 or placebo intravenously. No deaths or adverse events (AEs) leading to study discontinuation were reported. A summary of subject disposition is presented in Table 2. Two subjects from cohort B (0.66 mg/kg) were lost to follow-up. One subject who received placebo was lost to follow-up after day 2 and the other was lost to follow-up after day 57. The subject that was lost to follow-up after day 2 was replaced in cohort B at the time of cohort C (Fig. 1). A total of 18 research participants, six per cohort, received NTM-1634 (0.33 mg/kg, 0.66 mg/kg, or 1 mg/kg), and two research participants per cohort, six in total, received placebo. The doses were chosen to (i) deliver a dose of BoNT antitoxin that exceeded the current dose of BAT, (ii) achieve a serum concentration greater than the MAb *K_D_* for BoNT, and (iii) allow measurement of the concentration of each component MAb. For example, for a 70-kg subject, these doses would provide a total neutralizing capacity of 1.85 × 10^8^, 3.70 × 10^8^, and 5.6 × 10^8^ mouse LD_50_s of BoNT/C based on the preclinical data described above. This compares to a single dose BoNT/C neutralizing capacity for BAT of 3.0 × 10^7^ mouse LD_50_s.

**TABLE 2:** Summary of subject disposition (all subjects randomized)

Disposition	No. (%) treated with (mg/kg):				
	0.33 (*n* = 6)	0.66 (*n* = 6)	1 (*n* = 6)	Placebo (*n* = 7)	Total (*n* = 25)
All subjects randomized	6	6	6	7	25
Subjects who were dosed (safety population)	6 (100)	6 (100)	6 (100)	7 (100)	25
Subjects who completed study	6 (100)	5 (83)	6 (100)	6 (85.7)	23 (92)
Subjects who discontinued early from study	0	1 (16.7)	0	1 (14.3)	2 (8)
Lost to follow-up	0	1 (16.7)	0	1 (14.3)	2 (8)

The study participants ranged in age from 19 to 44 years. The majority of study subjects were white (17 [68%]), of Hispanic or Latino background (13 [52%]), and female (19 [74%]). The demographic characteristics were similar across the treatment groups except cohort C, where all the participants who received active compound were female. The demographic characteristics are summarized in Table 3.

**TABLE 3:** Summary of subject demographics

Parameter	Value for group treated with (mg/kg):				
	0.33 (*n* = 6)	0.66 (*n* = 6)	1 (*n* = 6)	Placebo (*n* = 7)	Total (*n* = 25)
Age (yr)					
Mean	33.7	30.0	34.7	32.9	32.8
SD	7.53	7.95	9.29	6.82	7.59
Minimum	25	19	21	23	19
Maximum	42	42	44	40	44
Sex (*n* [%])					
Male	2 (33.3)	2 (33.3)	0	2 (28.6)	6 (24.0)
Female	4 (66.7)	4 (66.7)	6 (100.0)	5 (71.4)	19 (76.0)
Ethnicity (*n* [%])					
Hispanic or Latino	4 (66.7)	3 (50.0)	3 (50.0)	3 (42.9)	13 (52.0)
Not Hispanic or Latino	2 (33.3)	3 (50.0)	3 (50.0)	4 (57.1)	12 (48.0)
Race (*n* [%])					
Black or African American	0	1 (16.7)	3 (50.0)	2 (28.6)	6 (24.0)
White	5 (83.3)	5 (83.3)	2 (33.3)	5 (71.4)	17 (68.0)
Other	1 (16.7)	0	1 (16.7)	0	2 (8.0)

### Safety profile.

No deaths or AEs leading to study discontinuation were reported. Overall, two non-drug-related serious adverse events (SAEs) were reported. One subject in cohort B (0.66 mg/kg) had an SAE of exacerbation of schizophrenia and one subject in cohort C (1 mg/kg) had and SAE of wrist fracture. Treatment-emergent adverse events (TEAEs) were reported in 19 of 25 subjects (76%), with a total of 69 TEAEs reported over the course of the study (Table 4). Cohort A (0.33 mg/kg) had four of six (66.7%) reporting 18 TEAEs. Cohort B (0.66 mg/kg) had five of six (83.3%) reporting 21 TEAEs. Cohort C (1 mg/kg) had six of six (100%) subjects reporting 20 TEAEs. The placebo group had four of seven (57.1%) subjects reporting 10 TEAEs. The most frequently reported TEAE was blood creatine phosphokinase increase, which occurred in 5 (20%) of the subjects. The next most frequently reported TEAEs (reported in two or more subjects overall) were hemoglobin decreases, hematuria, hematocrit decreases, viral upper respiratory tract infection, white blood cell count decreases, blood calcium decreases, blood potassium increases, blood sodium increases, cough, hypernatremia, myalgia, neutrophil count decreases, and proteinuria. There were no clinically significant abnormal electrocardiograms (ECGs) nor were there clinically meaningful trends identified comparing baseline vital and ECG values to those at subsequent time points. All TEAEs were considered by the investigator to be “not related” to the study drug. Antidrug antibody (ADA) results were negative for all participants with the exception of one subject who had a positive ADA result with a titer of 1.2 ng/ml for MAb XCD-b predose on day 1. All postdose ADA results were negative for this participant. The significance of this finding is unknown, but it may be due to the setting of the cut point for ADA detection. The subject did not report any past medical history and experienced no AEs during the study.

**TABLE 4:** Summary of treatment-emergent adverse events reported

TEAE	0.33 mg/kg (*n* = 6)		0.66 mg/kg (*n* = 6)		1 mg/kg (*n* = 6)		All active (*n* = 18)		Placebo (*n* = 7)		Total (*n* = 25)	
	No. (%) of subjects	No. of events	No. (%) of subjects	No. of events	No. (%) of subjects	No. of events	No. (%) of subjects	No. of events	No. (%) of subjects	No. of events	No. (%) of subjects	No. of events
Blood creatine phosphokinase increased	1 (16.7)	1	1 (16.7)	1	3 (50.0)	3	5 (27.8)	5	0	0	5 (20.0)	5
Hemoglobin decreased	1 (16.7)	1	0	0	2 (33.3)	2	3 (16.7)	3	1 (14.3)	1	4 (16.0)	4
Hematuria	0	0	0	0	1 (16.7)	1	1 (5.6)	1	2 (28.6)	3	3 (12.0)	4
Hematocrit decreased	1 (16.7)	1	0	0	2 (33.3)	2	3 (16.7)	3	0	0	3 (12.0)	3
Viral upper respiratory tract infection	2 (33.3)	2	1 (16.7)	1	0	0	3 (16.7)	3	0	0	3 (12.0)	3
White blood cell count decreased	1 (16.7)	1	1 (16.7)	1	1 (16.7)	1	3 (16.7)	3	0	0	3 (12.0)	3
Blood calcium decreased	1 (16.7)	1	0	0	0	0	1 (5.6)	1	1 (14.3)	1	2 (8.0)	2
Blood potassium increased	2 (33.3)	2	0	0	0	0	2 (11.1)	2	0	0	2 (8.0)	2
Blood sodium increased	1 (16.7)	1	0	0	0	0	1 (5.6)	1	1 (14.3)	1	2 (8.0)	2
Cough	0	0	1 (16.7)	1	1 (16.7)	1	2 (11.1)	2	0	0	2 (8.0)	2
Hypernatremia	0	0	2 (33.3)	2	0	0	2 (11.1)	2	0	0	2 (8.0)	2
Myalgia	1 (16.7)	1	0	0	1 (16.7)	1	2 (11.1)	2	0	0	2 (8.0)	2
Neutrophil count decreased	0	0	1 (16.7)	1	1 (16.7)	1	2 (11.1)	2	0	0	2 (8.0)	2
Proteinuria	0	0	0	0	0	0	0	0	2 (28.6)	2	2 (8.0)	2
												
Total TEAEs		11		7		12				8		38

### Pharmacokinetics analysis.

**FIG 2:**
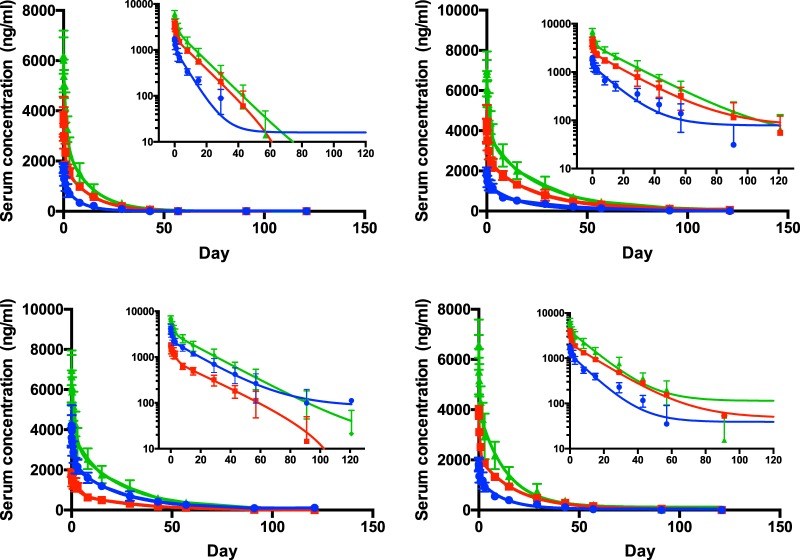
Serum concentration over time means from 6 subjects (error bars indicate standard deviations) at three dosing levels: 0.33 mg/kg (blue), 0.66 mg/kg (red), and 1 mg/kg (green). Curves are least-square best fit; for all curves, *R*^2^ ≥ 0.9. Curves are least-squared fits and do not converge at zero antibody concentration in log scale as a result of the lower limit of quantitation of the assay being 10 ng/ml and the lack of sampling after 150 days. (A) MAb XCD-a. (B) XCD-b. (C) XCD-c. (D) XCD-d.

A summary of the pharmacokinetics data is presented in Table 5. Peak concentrations for each of the antibodies, regardless of dose, were generally observed 1 to 2 h after the 1-h infusion. The peak concentrations of each of the four antibodies were also similar. After the peak, the concentrations of all four antibodies declined in a log-linear fashion, with a distinct distribution and terminal elimination phase (Fig. 2). Serum concentrations for XCD-a were quantifiable up to day 29 in 5 of 6 subjects, day 43 in 3 of 6 subjects, and day 57 in 1 of 6 subjects who received NTM-1634 0.33, 0.66, and 1 mg/kg, respectively. Serum concentrations of XCD-b were quantifiable up to day 91 in 2 of 6 subjects, day 121 in 2 of 6 subjects, and day 121 in 3 of 6 subjects who received NTM-1634 0.33, 0.66, and 1 mg/kg, respectively. Serum concentrations of XCD-c were quantifiable up to day 91 in 1 of 6 subjects, day 121 in 1 of 6 subjects, and day 121 in 1 of 6 subjects who received NTM-1634 0.33, 0.66, and 1 mg/kg, respectively. Serum concentrations of XCD-d were quantifiable up to day 57 in 2 of 6 subjects, day 91 in 2 of 6 subjects, and day 91 in 1 of 6 subjects who received NTM-1634 0.33, 0.66, and 1 mg/kg, respectively.

**TABLE 5:** Summary of pharmacokinetics data

MAb	Cohort (dose [mg/kg])	*C*_max_ (ng/ml)/GeoCV%	*T*_max_ (h [min, max])	AUC_0–t_ (μg·h/ml)/GeoCV%	AUC_0–∞_ (μg·h/ml)/GeoCV%	*t*_1/2_ (days)/GeoCV%
XCD-a	A (0.33)	1,762/11.2	1.00 (1.00, 2.00)	207/16.4	2,745/8.8	10.9/11.4
	B (0.66)	3,805/19.3	1.50 (1.00, 2.00)	525/28.1	635/11.1	10.6/17.5
	C (1)	6,347/14.0	2.00 (1.00, 4.02)	961/14.3	1,001/14.2	10.2/9.3
XCD-b	A (0.33)	1,969/10.7	2.00 (1.00, 2.08)	591/28.9	739/17.5	24.3/16.6
	B (0.66)	4,296/21.3	1.00 (1.00, 2.00)	1,530/21.4	1,674/21.3	24/28.4
	C (1)	7,226/10.2	2.00 (1.00, 4.00)	2,508/14.1	2,612/14.3	23.4/15.0
XCD-c	A (0.33)	1,877/10.0	1.00 (1.00, 2.00)	542/22.6	624/22.3	20.2/18.1
	B (0.66)	4,149/23.6	1.00 (1.00, 2.00)	1,339/23.8	1,492/20.7	22.3/27.5
	C (1)	7,151/12.0	2.00 (1.00, 4.02)	2,193/15.9	2,281/16.3	21.4/14.9
XCD-d	A (0.33)	1,830/14.0	2.00 (2.00, 4.00)	403/15.6	463/17.8	15.9/19.1
	B (0.66)	4,080/17.7	1.00 (1.00, 4.00)	990/25.4	1,083/25.2	17.8/35.0
	C (1)	6,829/10.5	1.00 (1.00, 4.00)	1,553/15.6	1,662/13.3	15.8/16.4

The dose-adjusted maximum concentrations of drug in serum (*C*_max_s) for all four antibodies were similar across all three dose cohorts. In addition, the areas under the concentration-time curves from 0 to infinity (AUC_0–∞_s) for XCD-b and XCD-c were not different across all three dose cohorts, indicating dose proportionality over the 3-fold dose range. The areas under the concentration-time curves from 0 to infinity (AUC_0–∞_s) for XCD-a and XCD-d were lower than for XCD-b and XCD-c, with XCD-a having the lowest AUC_0–∞_ across all three dose cohorts. Each of the four MAb had long half-lives that varied from 10.2 to 24 days. XCD-b and XCD-c had longer half-lives (*t*_1/2_s) than either XCD-a or XCD-d, with XCD-a having the shortest *t*_1/2_ at 263 h. The basis for the differences in half-lives between MAbs is unclear, since the MAbs have similar pIs and have the same antibody constant regions. The overall pharmacokinetics of each of the four individual antibodies were roughly similar to each other, with the rank order of XCD-b having the longest half-life and AUC followed by XCD-c, XCD-d, and then XCD-a.

## DISCUSSION

BoNTs are classified by the Centers for Disease Control and Prevention (CDC) as one of the highest-risk threat agents for bioterrorism due to their extreme potency and lethality, ease of production and transport, and need for prolonged intensive care (3). Thus, the development of countermeasures for all seven serotypes, including BoNT/C and BoNT/D, is a high research priority. Both Iraq and the former Soviet Union produced BoNT for use as weapons, and at least three additional countries (Iran, North Korea, and Syria) have developed or are believed to be developing BoNT as instruments of mass destruction (3, 28, 29). Iraq produced 19,000 liters of concentrated BoNT, more than any other biothreat agent, of which 10,000 liters were weaponized in missile warheads or bombs. The 19,000 liters represent an amount of toxin capable of killing the world’s population three times over. The Japanese cult Aum Shinrikyo attempted to use BoNT for bioterrorism by dispersing toxin aerosols at multiple sites in Tokyo (3).

Botulism causes significant morbidity and mortality, and exposure of even a small number of civilians would paralyze the health care delivery system of any metropolitan area. Treatment of botulism requires prolonged hospitalization in an intensive care unit (ICU) and mechanical ventilation for up to 6 weeks. There are no current logistically feasible prophylactic agents available in the United States as medical countermeasures. Antitoxin is the only effective treatment for botulism and has been shown to reduce the duration of hospitalization, duration of mechanical ventilation, and cost of hospitalization (30). The current treatment for adult botulism is heptavalent (serotypes A to G) equine botulism antitoxin (BAT) (18). BAT is immunogenic, and hypersensitivity reactions have been reported, including serum sickness and asystole (18, 31).

As an alternative and potentially safer and more effective product, we have been developing serotype-specific human or humanized MAb combinations that stoichiometrically neutralize BoNT by eliciting first-pass clearance through the liver (20, 27). In addition, these antibodies are being used in diagnostic tests to identify the specific serotype of botulinum neurotoxin that was causing the symptoms and provide guidance for treatment (32, 33). This study was the first in-human assessment of NTM-1634, a four-MAb combination which potently neutralizes BoNT/C, C/D, D/C, and D in rodents. The results demonstrate that single escalating doses of NTM-1634 administered intravenously into healthy subjects were well tolerated and safe. In addition, these doses also demonstrated acceptable immunogenicity profiles, with little ADA detected over the dose ranges and duration of the study. No dose-related SAEs were observed, and the AEs that were observed between the three cohorts and placebo group were similar in frequency, character, and severity. These results indicate that NTM-1634 may be a safer product than the equine polyclonal antibody BAT (18).

Previous studies with NTM-1634 have demonstrated that the effectiveness of NTM-1634 in neutralizing BoNT C/D is dependent on the presence of all four antibodies (C. Garcia-Rodriquez et al, unpublished data). This is consistent with observations for other anti-BoNT antibody combinations in development (20, 24, 25). It therefore follows that the duration of effectiveness of the combination will be determined by the monoclonal antibody that falls below the minimal effective levels most rapidly. The terminal *t*_1/2_s were similar across the range of doses for each antibody. At all doses given, however, XCD-a was cleared more rapidly than the other three, with XCD-d being the next most rapidly cleared. However, all antibodies were detectable for a minimum of 4 weeks, with the most rapidly clearing MAb, XCD-a, having a serum half-life of 11 days. This contrasts with BAT, which is an F(ab′)_2_ product with short serum half-lives for BoNT/C and BoNT/D (30 and 7.5 h, respectively). The long half-life of NTM-1634 may reduce the likelihood of a relapse of botulism that has been reported with BAT (19).

While we have not directly compared the potency of NTM-1634 to BAT for BoNT/C or BoNT/D, the potency of a BoNT/A MAb combination (NTM-1631) under development is 400- to 600-fold more potent than BAT in mouse protection studies with BoNT/A1, and a three-MAb combination to BoNT/F more than 150 to 450 times more potent than BAT (25, 27) on a weight basis. The lowest dose of NTM-1634 studied (0.33 mg/kg) would deliver a total BoNT/C neutralizing capacity of 1.85 × 10^8^ mouse LD_50_s of BoNT/C based on the preclinical data described above. This compares to a single dose BoNT/C neutralizing capacity for BAT of 3.0 × 10^7^ mouse LD_50_s, or more than 6-fold more. This greater potency is expected, since polyclonal antisera rarely have more than 1% of the total IgG directed against the target antigen, compared to ∼100% for recombinant IgG. The combined safety, potency, and long half-life of NTM-1634 make it possible for potential intramuscular or subcutaneous administration after exposure and prior to the development of symptoms, which is not possible with BAT. These features would make NTM-1634 simpler to administer in a mass casualty scenario. The high potency and long half-life would also permit administration of NTM-1634 for the prevention of botulism as an alternative to vaccination. This is important, since there is no longer a vaccine available to prevent types C and D botulism.

In summary, this study provides an early evaluation of the pharmacokinetics and safety profile of NTM-1634. The highly potent protection in animal models and the safety profile and long half-life of >1 month in humans demonstrate the potential utility of NTM-1634 for treatment of botulism due to serotypes C and D. Thus, further clinical development of NTM-1634 for the treatment and prevention of BoNT intoxication due to serotypes C and D is warranted.

## MATERIALS AND METHODS

This was a phase 1, single-center, placebo-controlled, double-blind dose escalation study to evaluate the safety, pharmacokinetics characteristics, and immunogenicity of NTM-1634 in healthy adults. This first in-human study consisted of three cohorts (A, 0.33 mg/kg; B, 0.66 mg/kg; C, 1 mg/kg) of eight subjects each. Each subject received a single i.v. infusion of NTM-1634 or placebo administered over 1 h. The placebo was normal saline.

### NTM-1634.

NTM-1634 consists of four human single-chain Fv (scFv) MAbs isolated from yeast display libraries constructed from the antibody variable region genes of humans immunized with pentavalent botulinum toxoid (C. Garcia-Rodriquez et al, in preparation). The heavy- and light-chain variable region genes of the scFv were inserted into vectors containing the human kappa and IgG1 constant region genes to create human IgG1/kappa MAbs, and four separate stable CHO-K1 cell lines were established. The IgG1 isotype was selected on the basis of studies showing the importance of Fc receptor engagement for the synergy in potency seen when the MAbs are combined (J. D. Marks, unpublished data). Each MAb (XCD-a, XCD-b, XCD-c, and XCD-d) was expressed and purified individually using protein A and ion-exchange chromatography and then combined in equimolar amounts to create NTM-1634.

### Subjects.

The study enrolled a total of 25 healthy adult volunteers. Subjects in all cohorts participated in the study for approximately 21 weeks, including a 4-week screening period, a 3-day inpatient stay, and approximately 17-week follow-up study after drug administration. Eligible research participants were healthy male or healthy, nonpregnant, nonlactating females between the ages of 18 and 45 years. Subjects had body mass indices of between 18.5 and 30 kg/m^2^, negative illicit drug screens, and adequate venous access for the infusion. Subjects were considered study ineligible if they had a history of a chronic medical condition that would interfere with the accurate assessment of the objectives of the study, a history of severe allergic reaction to any type of medications, bee stings, food, or environmental factor or reaction to immunoglobulins, positive serology for HIV, HBsAg, or hepatitis C virus (HCV) antibodies, were pregnant or breastfeeding, were previously exposed to BoNT and active drug, or have alcohol dependence. Research subjects who had received any monoclonal antibody in the past or any antibody or blood products, treatment with another investigational drug within 28 days of dosing, donated blood within 56 days of enrollment, or use of H1 antihistamines or beta-blockers within 5 days of dosing were also excluded. Additional exclusion criteria included a marked baseline prolongation of QT/QTc interval, clinically significant electrocardiogram, systolic blood pressure of >140 mm Hg or diastolic blood pressure >90 mm Hg, resting heart rate <50 or >100 beats per min, or oral temperature of ≥38°C.

### Study design.

Subjects were randomized into three cohorts of eight subjects and admitted to the study unit on day −1. Infusion occurred on day 1, and subjects remained at the clinic until discharge on day 2 (25 h after the infusion had ended). Follow-up visits occurred on days 3, 4, 8, 15, 29, 43, 57, 91, and 121. Safety parameters included physical examination, 12-lead ECG, vital signs, and clinical laboratory values. Pharmacokinetics samples were collected predose, at the end of infusion, 1, 3, 7, 23, 47, and 71 h postinfusion, and on days 8, 15, 29, 43, 57, 91, and 121 for all cohorts. Dose escalation did not occur until safety data from at least 7 subjects per cohort through day 8 were reviewed by the Safety Review Committee comprising the principal investigator (D. M. Snow), Ology Bioservices Medical Representative (C. N. Kraus), and the NIAID Division of Microbiology and Infectious Diseases Medical Monitor.

For each dosing cohort, the first two subjects were randomized 1:1 active/placebo so that one of the first two subjects received active treatment and the other control. The treatment assignment of the remaining six subjects in each cohort was 3:1 active/placebo. The randomization list was generated by the unblinded study biostatistician and transferred to the unblinded study pharmacist prior to the start of the study. The study staff participating in the administration of the study product and assessment of subjects were not aware of the contents of the i.v. bag. Drug and placebo appeared identical.

The study drug was manufactured by XOMA Corporation (Berkeley, CA) and filled by Althea Technologies (San Diego, CA). Assays developed for NTM-1643 characterization and release were qualified using biological testing, including a MAb-specific enzyme-linked immunosorbent binding assay (ELISA) which used recombinant BoNT/C protein domains (22, 34 and G. Manzanarez et al., unpublished data), developed and qualified by Ology Bioservices, and an *in vivo* quadruple antibody protection assay in mice, developed and qualified in collaboration with SRI International (Menlo Park, CA). The investigational product was formulated as a 5-mg/ml, clear colorless, sterile aqueous solution in a pH 6.0 buffered vehicle without any preservatives. The drug product was supplied in 2-ml, pyrogen-free, type 1 glass vials. Placebo was a sterile, nonpyrogenic isotonic solution of 0.9% sodium chloride injection, USP grade, and water for injection. The placebo did not contain preservatives, bacteriostatic or antimicrobial agents, or added buffer. Normal saline was used to dilute the NTM-1634 for i.v. infusion and was supplied in single-dose plastic containers.

### Safety analyses.

The safety endpoints were the occurrence of serious adverse events (SAEs) following administration of NTM-1634 to the final follow-up visit, the occurrence of adverse events (AEs) from administration of NTM-1634 to day 57, the occurrence of changes from baseline in physical examination, vital signs, and clinical safety laboratory values following administration of NTM-1634 to the final follow-up visit, and the occurrence of changes from baseline in ECG parameters after administration of NTM-1634 on day 1 (day of infusion). Immunogenicity was assessed by determining the presence of human anti-human antibodies.

### Pharmacokinetics analyses.

Four immunoassays were developed to measure the concentrations of XCD-a, XCD-b, XCD-c, and XCD-d in serum samples. A bridging electrochemiluminescence (ECL) assay was used (22, 27, 34). Biotinylated (b) and ruthenylated (ru) BoNT/C domains were used in the assay as the capturing and detecting reagents, respectively. The assay uses the bivalent binding capability of the antibodies to form a bridging complex with biotinylated-domain and ruthenylated-domain to generate ECL signals for the measurement of the target antibody concentration in serum. Six assays were developed using the same format for each antibody assay. ECL signals generated from captured immune complexes formed when one arm of the MAb bound to a biotinylated domain and the other arm to the ruthenylated domain were detected by a Meso-Scale Discovery (MSD)SECTOR Imager 6000 and reported in ECL units. Calibration standards and quality control samples were prepared by spiking known amounts of each MAb into human serum. With the minimum required dilution of 1:10, the lower limit of quantitation (LLOQ) was 10 ng/ml, with a quantifiable range of 10 to 2,000 ng/ml.

Immunogenicity assays were developed and validated to detect anti-XCD-a, XCD-b, XCD-c, and XCD-d antibodies in human serum. Bridging ECL assays used labeled MAbs for the capture and detection of antidrug antibodies (ADAs) in serum. A mixture of biotinylated and ruthenylated XCD-a, XCD-b, XCD-c, and XCD-d MAbs were incubated with human serum, and ECL signals were generated when one arm of an ADA bound to a b-MAb and the other arm bound to a ru-MAb. Samples that generated a signal above the screening cut point (SCP) were considered positive and confirmed with the use of a competitive assay in which the serum samples were incubated with and without nonlabeled MAbs. Samples with ECL signals above the confirmation cut point were considered positive and further analyzed for titers.

### Statistical analyses.

Pharmacokinetics (PK) parameters for each of the four monoclonal antibodies of NTM-1634 (XCD-a, XCD-b, XCD-c, and XCD-d) were calculated from the serum concentration-time data using noncompartmental techniques (Phoenix-WinNonlin version 6.3.0; Pharsight Corporation, St. Louis, MO) and actual sampling times based on the start of the infusion. The following PK parameters were reported: area under the concentration time-curve (AUC) to the last concentration above the lower limit of quantitation (AUC_0–t_), maximum observed concentration (*C*_max_), time that *C*_max_ was observed (*T*_max_), terminal elimination rate constant (λ_z_), AUC from time zero extrapolated to infinity (AUC_0–∞_), terminal elimination half-life (*t*_1/2_), total clearance (CL), and volume of distribution (*V*_z_). Summary of *T*_max_ included the number of non-missing observations (*n*), median, minimum, and maximum values only. For all other PK parameters and concentrations, *n*, mean, standard deviation (SD), median, minimum, and maximum values are presented. In addition, geometric mean (GeoMean) and geometric coefficient of variation (GeoCV%) are presented for serum concentrations, *C*_max_, AUC_0–t_, AUC_0–∞_, *t*_1/2_, CL, and *V*_z_.

The overall incidence of treatment-emergent AEs (TEAEs) (number and percentage of subjects) was summarized by dose cohort, and overall for categories of degree of severity, SAEs, causally related TEAEs, and SAEs by causality. The TEAEs were summarized and tabulated at both the subject (number [percentage] of subjects) and event (number of events) level for each dose cohort and overall. Treatment-emergent laboratory abnormalities by toxicity grades are summarized for each cohort and overall. Observed values and change from baseline of continuous ECG measurements (heart rate, QRS duration [milliseconds], PR interval [milliseconds], QT interval [milliseconds], and QTcF interval [milliseconds]) are summarized by cohort and overall. The overall ECG interpretation is summarized as “normal,” “abnormal, not clinically significant,” and “abnormal, clinically significant.”
